# Early Alzheimer's and Parkinson's Disease Pathology in Urban Children: Friend versus Foe Responses—It Is Time to Face the Evidence

**DOI:** 10.1155/2013/161687

**Published:** 2013-02-07

**Authors:** Lilian Calderón-Garcidueñas, Maricela Franco-Lira, Antonieta Mora-Tiscareño, Humberto Medina-Cortina, Ricardo Torres-Jardón, Michael Kavanaugh

**Affiliations:** ^1^Center for Structural and Functional Neurosciences, The University of Montana, 32 Campus Drive, Skaggs Building 287, Missoula, MT 59812, USA; ^2^Departamento de Investigación, Hospital Central Militar, Secretaria de la Defensa Nacional, 11649 México, DF, Mexico; ^3^Departamentos de Radiología y Patología Experimental, Instituto Nacional de Pediatria, 04530 México, DF, Mexico; ^4^Centro de Ciencias de la Atmósfera, Universidad Nacional Autónoma de México, 04510 México, DF, Mexico

## Abstract

Chronic exposure to particulate matter air pollution is known to cause inflammation leading to respiratory- and cardiovascular-related sickness and death. Mexico City Metropolitan Area children exhibit an early brain imbalance in genes involved in oxidative stress, inflammation, and innate and adaptive immune responses. Early dysregulated neuroinflammation, brain microvascular damage, production of potent vasoconstrictors, and perturbations in the integrity of the neurovascular unit likely contribute to progressive neurodegenerative processes. The accumulation of misfolded proteins coincides with the anatomical distribution observed in the early stages of both Alzheimer's and Parkinson's diseases. We contend misfolding of hyperphosphorylated tau (HP**π**), alpha-synuclein, and beta-amyloid could represent a compensatory early protective response to the sustained systemic and brain inflammation. However, we favor the view that the chronic systemic and brain dysregulated inflammation and the diffuse vascular damage contribute to the establishment of neurodegenerative processes with childhood clinical manifestations. Friend turns Foe early; therefore, implementation of neuroprotective measures to ameliorate or stop the inflammatory and neurodegenerative processes is warranted in exposed children. Epidemiological, cognitive, structural, and functional neuroimaging and mechanistic studies into the association between air pollution exposures and the development of neuroinflammation and neurodegeneration in children are of pressing importance for public health.

## 1. Introduction

Air pollution is a significant health problem in megacities around the world [1–3]. In a scenario where the projected world population will have a further increase of 2 to 4.5 billion in the first 50 years of this century [4], the issue of deteriorating environments and their health impact is critical. The problem of air pollution is not confined to large urban centers, it also affects small cities and rural areas. Particulate matter (PM) air pollution is a public health problem affecting millions of people worldwide.

Recent works have shed new light on the etiology of Alzheimer's and Parkinson's diseases (AD and PD), with a growing body of evidence that oxidative stress and neuroinflammation are at the core of their etiopathogenesis and that there is a close interplay between environmental factors and neurodegeneration [5–8]. We also know the most beneficial neuroprotective effects might only be achieved in the very early stages of the detrimental processes. As such, a great effort has been made in establishing the associations between particulate air pollution, neuroinflammation, and neurodegeneration in highly exposed megacity children and young adults. The first part of this paper deals briefly with the current state of air pollution in Mexico City Metropolitan Area (MCMA) and with the several areas of investigation in our laboratory that exemplify how seemingly clinically healthy children are responding to the sustained exposures to air pollutants. The second part of the paper turns to a more troublesome challenge. How do you formulate the neuropathology and gene brain expression findings in *clinically healthy children and young adults* and establish the links with the current mainstream concepts of neurodegeneration. This principled problem thus addresses the relation between neuroinflammation, neurodegeneration, and air pollution exposures with an emphasis on compensatory responses. Dealing with this problem invites the development of linking hypotheses between the domains and the need for intervention, issues addressed in the third part of the paper. 

## 2. Air Pollution Background and Clinical Issues in Metropolitan Mexico City Clinically Health Children

### 2.1. Air Pollution in Mexico City Metropolitan Area (MCMA)

Although there is significant air pollution associated with ozone in MCMA, in this work, we will focus on particulate matter (PM) broadly defined by the diameter of the aerodynamic particles and classified into coarse particles (<10 *μ*m; PM_10_), fine particles (<2.5 *μ*m; PM_2.5_), and ultrafine particles (<100 nm; UFPM). Fine and ultrafine PM are of particular interest given their capability to reach the brain [9]. The smaller the particle, the greater its penetration, diffusion, and deposition into the respiratory tract and its direct translocation into the brain [9–11]. 

MCMA, the largest urban center in North America, is an example of extreme urban growth and environmental pollution [12]. The metropolitan area of over 2000 square kilometers is home to over 20 million inhabitants including 8 million children. The energy demand of this population and over 40000 industries and 4 million vehicles consumes more than 40 million liters of petroleum fuels per day resulting in an annual emission of approximately 2.6 tons of pollutants including coarse and fine particulate matter, gaseous pollutants, polycyclic aromatic hydrocarbons, and lipopolysaccharides [12]. The MCMA is located in the southwestern portion of an elevated basin 2240 m above sea level that is surrounded on three sides by mountain ridges at 19° N 99° W. The high altitude and tropical insolation of the basin facilitate ozone production all year and contribute to the formation of secondary PM. Air quality is generally worse in the winter when thermal inversions are more frequent [13]. 

Even with the substantial reductions in the concentrations of some criteria pollutants (such as lead, CO, and SO_2_) achieved during the past fifteen years, MCMA residents remain exposed to concentrations of airborne pollutants exceeding current ambient air quality standards for PM and ozone [14]. High concentrations of PM_2.5_ as well as significant levels of PM_10_ associated with lipopolysaccharides (PM-LPS) have been registered historically in Mexico City's air, and marked regional differences in the air pollutants concentrations and composition have been reported within MCMA [12, 15–19]. 

**Figure 1:**
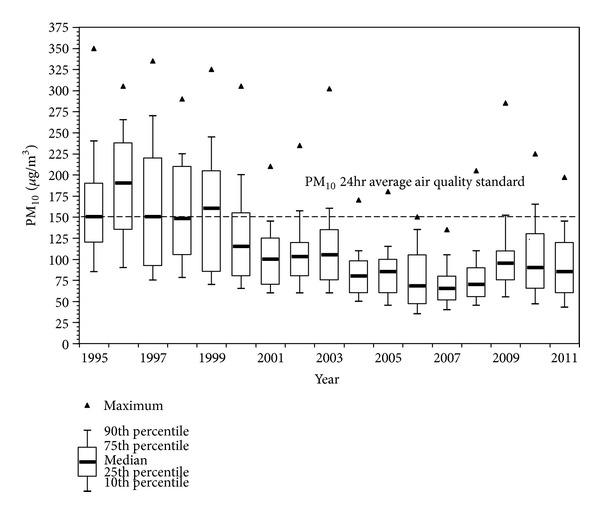
Trend of the PM_10_ 24-hour average concentrations from all monitoring stations in the MCMA from 1995 to 2011. The dashed line shows the U.S. EPA PM_10_ 24 hr average air quality standard (data from the SMA-GDF).

**Figure 2:**
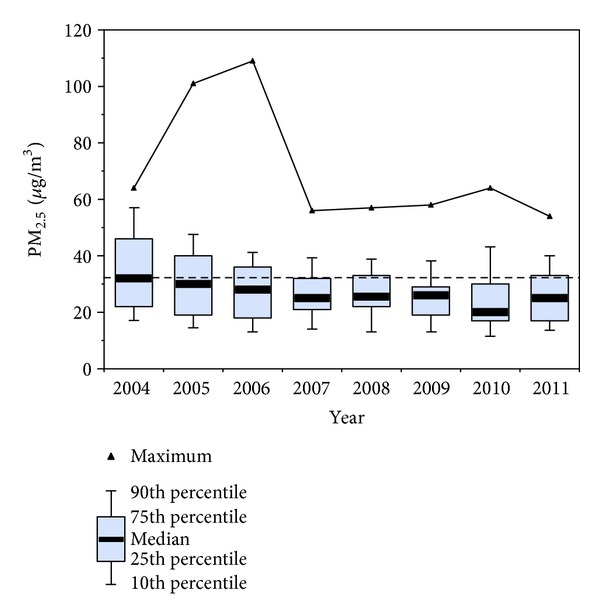
Trend of the PM_2.5_ 24-hour average concentrations from all monitoring stations in the MCMA from 2004 to 2011. The dashed line shows the U.S. EPA PM_2.5_ 24 hr average air quality standard (data from the SMA-GDF).

Figure 1 shows the trend of 24-hour average PM_10_ concentrations for MCMA (1995–2011). PM_10_ concentrations had shown a clear reduction up to 2007; however, concentrations have been slowly back on the rise in the last 5 years. PM_2.5_ data from the monitoring network [20] in Figure 2 show the 90th percentile of the 24-hour average concentrations per year have been above the respective air quality standard of 35 *μ*g/m^3^. MCMA residents are also exposed to UFPM from ambient air and workplaces. These nano sized PM include combustion sources (e.g., diesel exhaust particles, welding fumes) and manufactured or engineered nanoparticles (NPs). It is not widely appreciated that nano-sized materials are also present in many consumer products to which large segments of the population are exposed (e.g., toothpastes, cosmetics, sunscreens, food additives, and laser printer emissions) [21, 22].

In this massive exposure chamber, 8 million children and teens <18 y are receiving the impact of the involuntary exposure to the polluted air. 

### 2.2. Detrimental NonCNS Effects in Exposed Children

It is important to emphasize that PM exposure has been epidemiologically associated to a wide spectrum of cardiovascular, pulmonary, and CNS effects [10, 11, 23–25]. Exposure to fine PM over a few hours to weeks can trigger cardiovascular disease-related mortality and nonfatal events [10]. Longer-term exposure increases the risk for cardiovascular mortality to an even greater extent than exposures over a few days [10]. In the cardiovascular literature “*credible pathological mechanisms have been elucidated that lend biological plausibility to* (detrimental)* findings*” [10]. Two mechanistic pathways applied to the cardiovascular and lung effects fit precisely the detrimental pathways in place in MCMA children [8]. These pathways include: pulmonary and systemic oxidative stress, and inflammation and direct effects of PM or its constituents on the vasculature and/or blood elements after translocation from the lung [10]. 

The pediatric studies from our laboratory cited in this work were performed in Mexico City clinically healthy children with no known risk factors for pulmonary, cardiovascular, and CNS pathology or cognitive deficits. MCMA children are selected from nonsmoking families and their results compared to age, gender, and socioeconomic status (SES) matched children residing in low polluted places. The detrimental nonCNS effects associated to residency in MC include the following.

- Systemic inflammation with increased concentrations of proinflammatory cytokines, chemokines, and potent vasoconstrictors (i.e., endothelin-1, ET-1). The concentrations of inflammatory mediators and ET-1 correlate positively with cumulative exposures to PM_2.5_ and outdoor exposure hours [26]. Chronic inflammation involving the upper and lower respiratory tracts has been identified as a link between air pollution and brain damage [26–33]. Continuous expression of inflammatory mediators capable of reaching the CNS promotes the formation of reactive oxygen species (ROS) [8]. Activation of innate immune responses within the brain may follow the interactions between circulating cytokines and the constitutively expressed cytokine receptors of brain endothelial cells. Such responses may, in turn, be followed by activation of cells involved in adaptive immunity [30, 34, 35]. Monocytes are the main innate immune response mediator cells, producing and secreting TNF-*α*, interleukin-6 (IL-6), and IL-1*β*, which in turn recruit and increase the activity of other immune cells [34]. Sustained exposures to fine and ultrafine PM likely start a chain of events leading to brain endothelial cell activation, disruption of the neurovascular unit, altered response of the innate immune system, neuroinflammation, and neurodegeneration [8, 30, 34–36].
- Altered immune responses include significant decreases in the numbers of natural killer cells and increased numbers of mCD14+ monocytes and CD8+ cells. The reduction in the number of NK cells goes along with the low concentrations of interferon gamma (IFN-*γ*) [28]. MCMA children have monocytic mCD14 upregulation—a key membranous receptor involved in lipopolysaccharide (LPS) binding. The CD14 upregulation represents the early step in cell activation by LPS involving the innate immune initial host response to Gram negative bacterial infections [37]. MCMA children are historically exposed to endotoxin associated with PM [17, 28, 32, 38]. The issue is very important because we have shown there is a significant frontal upregulation of inflammasome-associated genes in MCMA children and young adults [30]. Moreover, particle exposure has been associated to pathogen sensors and the signaling by ROS drives inflammasome intracellular signaling complexes activation [39–41]. Even very low doses of LPS elicit an augmented response to subsequent endotoxin challenge with a violent immune response [42]. The priming phenomenon could play a role in the neuroinflammatory responses observed in MCMA children [30, 35].
- Pulmonary changes in MCMA children living in tobacco free homes include bilateral hyperinflation and increased linear markings observed in chest radiographs and mild bronchial wall thickening, prominent central airways, air trapping and pulmonary nodules identified by computed tomography scans. Abnormal lung function tests based on predicted values are seen in 7.8% of MCMA children. Higher concentrations of endothelin-1 correlate with elevations of mean pulmonary artery pressure, average hours per day spent outdoors, and 7 day cumulative concentrations of fine PM_2.5_ [26, 27].
- Cardiovascular effects include a significant right ventricle upregulation of IL-1*β*, TNF-*α*, IL-10, and CD14, and a left ventricle difference in TNF-*α*, and IL-10 in South versus North Mexico City residents, a key point in relation to the marked difference in pollutant profiles determined by the residence MCMA location [43].

### 2.3. Detrimental CNS Effects in Exposed Children

MCMA children with no known risk factors for neurological or cognitive disorders exhibit significant deficits in a combination of fluid and crystallized cognition tasks versus control children [29]. Fifty-six percent of MCMA children showed prefrontal white matter hyperintense (WMH) lesions by MRI and similar lesions were observed in MCMA dogs (57%) [29]. One control child out of 13 tested exhibited a single white matter lesion, and this child was an APOE 3/4 carrier [29]. Critical to this paper, MC breed animal facility dogs had frontal lesions with vascular subcortical pathology associated with neuroinflammation, enlarged Virchow-Robin spaces, gliosis, and ultrafine particulate matter deposition [29]. The dogs MRI findings were the same as the children, including their prefrontal location [29]. The data suggested the prefrontal cortex was a target anatomical region in exposed children and its damage could have contributed to their cognitive dysfunction. We next tested whether patterns of brain growth, cognitive deficits, and WMH were associated with exposures to MCMA air pollution [44]. Baseline and 1-year followup measurements of global and regional brain MRI volumes, cognitive abilities (Wechsler Intelligence Scale for Children-Revised, WISC-R), and serum inflammatory mediators were collected in 20 MCMA children (10 with white matter hyperintensities, WMH (+), and 10 without, WMH (−)) and 10 matched controls (CTL). There were significant differences in white matter volumes between CTL and MCMA children—both WMH (+) and WMH (−)—in right parietal and bilateral temporal areas. Both WMH (−) and WMH (+) MC children showed progressive deficits, compared to CTL children, on the WISC-R Vocabulary and Digit Span subtests. Interestingly, the cognitive deficits in MCMA children matched the localization of the volumetric differences detected over the 1 year followup [44]. 

When we analyzed the WMH lesions in relation to the profile of cytokines and chemokines [32], MCMA WMH (−) children displayed the profile of classical proinflammatory defensive responses: high interleukin 12, production of powerful proinflammatory cytokines, and low concentrations of key cytokines and chemokines associated with neuroprotection. In contrast, MC WMH (+) children exhibited a response involved in resolution of inflammation, immunoregulation, and tissue remodeling. The MC WMH (+) group responded to the air pollution-associated brain volumetric alterations with white and grey matter volume increases in temporal, parietal, and frontal regions and better cognitive performance compared to MC WMH (−). 

These findings suggest a complex modulation of cytokines and chemokines influencing children's white matter hyperintensities, volumetric white matter responses and cognitive outcomes as a result of environmental pollution exposures. 

## 3. Neuroinflammation and Neuropathology in Mexico City Children and Young Adults and Comparative Studies

In 2002, we published a dog study pointing to the nasal cavity as a major portal of entry of xenobiotics to the brain [45]. The study evaluated 32 healthy mongrel MCMA dogs, versus 8 dogs from Tlaxcala, a low polluted control city. MCMA dogs exhibited expression of nuclear neuronal NF-kappa B and iNOS in cortical endothelial cells at ages 2 and 4 weeks with subsequent damage to the blood-brain barrier (BBB), deposition of Apolipoprotein E (APOE)-positive lipid droplets in smooth muscle cells and pericytes, diffuse amyloid plaques, and neurofibrillary tangles [45]. Nasal respiratory and olfactory epithelium were clearly found to be early pollutant targets, as evidenced by the significant apurinic/apyrimidinic (AP) sites in MCMA dogs versus controls [46]. Moreover, olfactory bulb and hippocampal AP sites were also significantly higher in MCMA animals and nickel (Ni) and vanadium (V) were present in a gradient from olfactory mucosa > olfactory bulb > frontal cortex [46]. Striking findings in our canine studies included the presence of diffuse amyloid plaques in 11-month-old dogs and the presence of oil combustion PM-associated metals Ni and V in brain target areas. The dog studies are critical as they showed Alzheimer pathology beginning early in life with air pollutants playing a crucial role. Healthy young dogs exhibit a striking acceleration of Alzheimer's pathology when they live in a highly polluted place. It is well known that dogs are a good aging model and AD-type pathology and cognitive deficits are seen in older animals [47–49]. 

### 3.1. Neuroinflammation and Vascular Damage in MCMA Children and Young Adults

A very critical component of air pollution exposure is neuroinflammation [8, 50–52]. MCMA young urbanites exhibit an important frontal imbalance in genes essential for inflammation, innate and adaptive immune responses, oxidative stress, cell proliferation and apoptosis, when compared to age-matched residents in low pollution cities [30]. Measurements of mRNA cyclooxygenase-2, interleukin-1beta, and CD14 in target brain regions from 12 controls and 35 MC residents aged 25.1 ± 1.5 years showed upregulation of cyclooxygenase-2, IL-1*β*, and CD14 in supra, and infratentorial regions and cranial nerves including: olfactory bulb, frontal cortex, substantia nigrae, and the vagus nerve [35]. 

**Figure 3:**
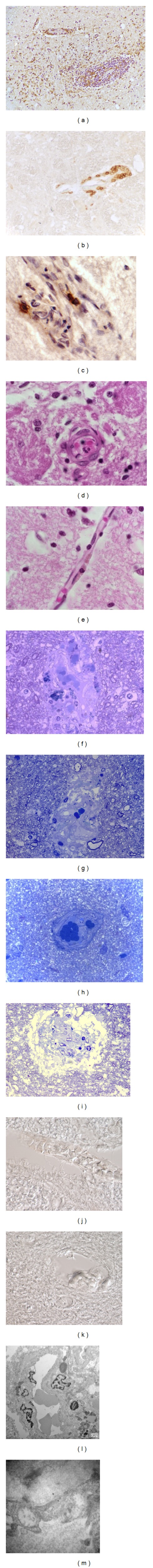
(a) Eleven year old MCMA girl with abundant ionized calcium binding adaptor molecule 1 (Iba-1)-positive microglia. Approximately 50% of the perivascular cells are Iba-1+ (Iba-1 antibody with DAB + brown product). (b) Eleven year old MCMA girl with brainstem perivascular accumulation of HLA-DR positive cells (HLA-DR antibody and DAB + brown product). (c) Frontal cortex in a MCMA 24 y old male with perivascular partially degranulated tryptase positive cells (Tryptase Ab with DAB + brown product). (d) Olfactory bulb blood vessel in a 14 year old MCMA boy. Notice a vacuolated endothelial cell and a polymorphonuclear leucocyte (PMN) attached to the vessel wall. Two glomeruli are adjacent to the damaged vessel. H&E. (e) Olfactory bulb blood vessel in a 14-year-old MCMA male APOE 3/3. Endothelial cells in the delicate vessel exhibit abundant lipofuscin, a highly oxidized and covalently cross-linked aggregate of proteins associated with aging. H&E. (f) Frontal cortex white matter from a MCMA 33 year old healthy subject with a cluster of blood vessels displaying perivascular numerous macrophages with lisosomal bodies and lipofuscin. The larger vessel displays abundant cell debris within the wall. One micron toluidine blue section. (g) Fourteen year old MCMA girl prefrontal white matter with an abnormal blood vessel displaying perivascular macrophages with lisosomal bodies and lipofuscin, abundant cell debri within the wall, apoptotic nuclei and focal enlargement of the Virchow-Robin space. One micron toluidine blue section. (h) Vascular lesions are also seen in young MCMA dogs. This 19 month dog exhibits a frontal white matter arteriole with hyperplastic endothelial cells partially reducing the lumen. One micron toluidine blue section. (i) The prefrontal cortex exhibits extensive vascular white matter damage, illustrated in this 13 y old MCMA girl. The arteriole shows extensive perivascular accumulation of macrophages with abundant lisosomal bodies. A striking enlargement of the Virchow-Robin space is seen with focal white matter damage. One micron toluidine blue section. (j) Seventeen year old MCMA teen brainstem blood vessel with extensive leaking expanding the Virchow-Robin space. (k) Same child as (j), the breakdown of the neurovascular unit also affects smaller blood vessels. (l) Electron micrograph of an olfactory bulb arteriole in a 17 y old MCMA boy. There is marked focal thickening of the vessel wall, numerous perivascular macrophages with lisosomal bodies and lipofuscin and vacuolization of endothelial cells. (m) Nanosized particles are seen in endothelial cells in many brain regions. This electron micrograph from a 17 y old MCMA male shows an arteriole in the olfactory bulb with two sharply defined particles in the endothelial cell cytoplasm and its basement membrane. The particles are 16 to 20 nanometers. EM ×50,000.

The entry of activated lymphocytes, mast cells, and macrophages into the brain parenchyma is a hallmark of chronic inflammatory processes [34, 53–56]. Clusters of mononuclear cells around blood vessels and activated microglia in the frontal and temporal cortex, subicular area, and the brain stem (Figure 3(a)) were present in all MCMA children and were extremely rare in control children [30, 35]. These mononuclear cells are positive for CD68, CD163, Iba-1 (Figure 3(a)), and HLA-DR (Figure 3(b)) [57]. Intact and degranulated mast cells identified by means of tryptase monoclonal antibodies are seen in perivascular locations in frontal (Figure 3(c)) and temporal cortices, as well in trigeminal ganglia, and in peripheral autonomic nerves innervating the lungs and hearts in MCMA subjects, whereas in the controls mast cells were rare and intact. Blood vessels exhibit vacuolated endothelial cells and marginal WBCs, both indicative of endothelial damage and activation (Figure 3(d)). While the presence of abundant lipofuscin in endothelial cells (Figure 3(e))—usually associated with aging and indicative of a highly oxidized and covalently cross-linked aggregate of proteins—is evidence of a dysfunctional lysosomal degradation not expected in children or young adults.

There was extensive vascular damage in the olfactory bulb and in the frontal cortex. In the prefrontal cortex, the vascular damage affects predominantly white matter (Figure 3(f)). The main vascular findings included thickened walls, abundant perivascular macrophages, and focal enlargement of the Virchow-Robin spaces (Figure 3(g)). Young dogs show similar lesions to children with significant endothelial cell hyperplasia markedly reducing the vessel lumen (Figure 3(h)). The extensive prefrontal vascular damage is accompanied by white matter focal damage that in some children is significant (Figure 3(i)). Extensive leaking of blood vessels involves supra and infratentorial regions (Figures 3(j) and 3(k)). Olfactory bulb arterioles also show marked focal thickening of the vessel walls, indicative of a chronic reparative process (Figure 3(l)). 

Ultrafine particles are likely players in the endothelial cell activation and are found in various CNS regions, including the Olfactory bulb (Figure 3(m)). UFPM are also seen in erythrocytes with the formation of patterned discrete contact points between endothelial cells and RBCs in the CNS, trigeminal ganglia, and lung capillaries of highly exposed people [35].

### 3.2. Alzheimer's and Parkinson's Diseases Hallmarks

**Figure 4:**
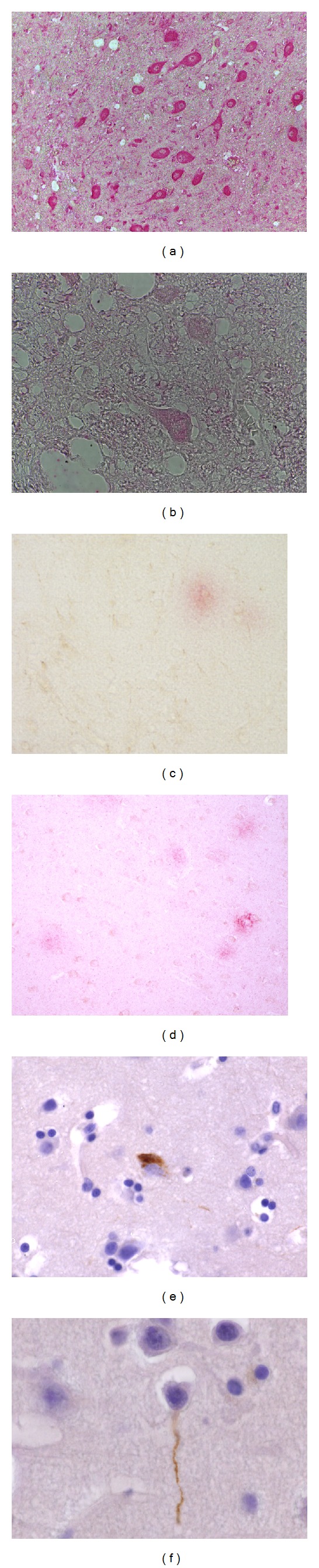
(a) Eleven year MCMA girl dorsal motor nucleus of the vagus stained with anti-8-OHdG showing immunohistochemical staining of oxidized nucleoside in neurons. 8-hydroxyguanosine is a modified base that occurs in DNA due to attack by hydroxyl radicals that are formed as byproducts and intermediates of aerobic metabolism and during oxidative stress. 8-OHdG immunohistochemistry red product. (b) Same 11 y old girl as in Figure 4(a) showing caudal pontine reticular nucleus neuronal protein oxidation marked by nitrotyrosine immunoreactivity. (c) Frontal cortex in an APOE 3/3 17 y old MCMA teen. A diffuse amyloid plaque (red product) is seen surrounded by glial cells negative for reactive astrocytes as detected by their reaction to the glial fibrillary acidic protein (GFAP). Dual immunohistochemistry for amyloid beta 1–42 and GFAP (DAB + brown product). (d) Frontal cortex in a 36 y old MCMA male APOE 3/4. This subject shows numerous diffuse and mature amyloid beta 1–42 plaques. (e) Frontal cortex in a 15 y old MCMA APOE 3/3 boy. Abnormal tau protein positive with the Tau 8 antibody (Innogenetics, Belgium), both in the neuronal body and in neuritis. (f) Frontal cortex in a 15 y old MCMA APOE 3/4 boy. A clear Tau 8 positive neurite is seen.

A growing body of epidemiologic and experimental data point to particulate matter components of air pollution as well as nanoparticles in the environment as risk factors for neurodegenerative diseases [51, 52, 58–63]. Indeed, exposure to different size and composition PM produce molecular hallmarks of neurodegeneration, including the production and deposit of misfolded protein aggregates (amyloid, alpha synuclein, hyperphosphorylated tau), oxidative stress, cell damage and death in susceptible neuronal populations [51, 52, 64–66]. Neuronal oxidative stress is prominent even in small MCMA children [35]. Extensive cytoplasmic accumulation of 8OHdG in key neuronal complexes (Figure 4(a)) correlates with oxidative stress and damage to DNA. Nitrotyrosine, a marker for inflammation and nitric oxide (NO) production, is also present in frontal neurons and infratentorial neuronal groups (Figure 4(b)). Nitrotyrosine positive inclusions are also seen in glial cells, microglia, and perivascular macrophages [35]. 

#### 3.2.1. Cortical Neurodegeneration Hallmarks

In young MCMA residents, amyloid beta42 (A*β*42) frontal (Figure 4(c)), olfactory bulb, and/or hippocampal immunoreactivity was observed in 58.8% of Apolipoprotein E (APOE) 3/3 <25 y, and 100% of the APOE 4 subjects (Figure 4(d)), whereas *α*-synuclein was seen in 23.5% of <25 y subjects [29]. In a different MCMA cohort, aged 18.3 ± 6.9 years, 40% exhibited tau hyperphosphorylation with pretangle material (Figures 4(e) and 4(f)) and 51% had A*β*42 diffuse frontal plaques compared with 0% in controls [30]. Thus, diffuse amyloid plaques and pretangle hyperphosphorilated tau are common frontal findings in highly exposed children, while low pollution controls are negative.

#### 3.2.2. Brainstem Neurodegeneration Hallmarks

**Figure 5:**
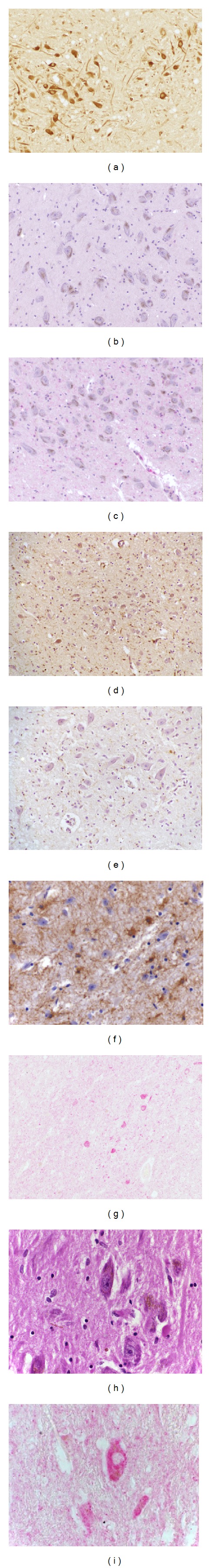
(a) Medial superior olive neurons from an 11 year old MCMA girl exhibit strong positivity for 8-hydroxyguanosine indicative of oxidative stress. 8-OHdG immunohistochemistry DAB brown product. (b) Substantia nigrae, pars compacta in a 17 y old Control teen. The section has been stained for the ionized calcium binding adaptor molecule 1 (IBA-1). There are very few positive cells. IBA-1 antibody with red product. (c) In contrast, this is the substantia nigrae, pars compacta in a 14 y old MCMA teen stained for IBA-1. Numerous positive cells are seen among the pigmented neurons and in perivascular locations. IBA-1 antibody with red product. (d) The same child has numerous positive IBA-1 activated microglia in her vestibular nuclei. IBA-1 antibody with brown product. (e) Same child as previous picture. Positive IBA-1 activated microglia in her dorsal motor nucleus of the vagus. IBA-1 antibody with brown product. (f) Reactive astrocytes are part of the response of glial cells to cell damage. Reactive astrocytes positive for GFAP surround the dorsal motor nucleus of the vagus neurons in this MCMA teen. GFAP with DAB brown product. (f) The dorsal motor nucleus of the vagus displays positive *α* synuclein neurons in the same child as 5F. *α*-Synuclein with red product. (h) The substantia nigrae is an early target in highly exposed teens. In this 11 y old girl there are partially degranulated pigmented neurons with a few macrophages containing the pigmented granules. An elongated microglia-like cell contains such brown granules in the vicinity of a neuronal shadow. H&E. (i) Substantia nigrae pigmented neuron is positive for alpha-synuclein in this 14 y old MCMA girl. *α*-Synuclein with red product.

Infratentorial involvement is also present in exposed children thus neuropathology is seen in the brainstems of children age 96.3 ± 8.5 months from highly polluted (*n* = 34) versus a low polluted city (*n* = 17) [67].  Figure 5(a) shows medial superior olivary neurons with strong oxidative stress as evidenced by their 8-hydroxyguanosine immunoreactivity. MC children have auditory and vestibular abnormal findings [67]. The pathology involves every level of the brainstem from the midbrain to the lower medulla. The substantia nigrae pars compacta displays IBA-1 microglia. The number of activated microglia also varies significantly between control and MCMA children (Figures 5(b) and 5(c)). Activated microglia are found throughout the brainstem in exposed children (Figures 5(c), 5(d), and 5(e)), along with reactive glial fibrillary acidic protein (GFAP) positive astrocytes, indicative of responsive glia to cell damage (Figure 5(f)). Accumulation of *α*-synuclein, activated microglia, extracellular neuromelanin, and pigment-laden macrophages are seen from the dorsal motor nucleus of the vagus level (Figure 5(g)) to the substantia nigrae midbrain sections (Figures 5(h) and 5(i)). There is a punctuated cytoplasmic accumulation of *α*-synuclein in affected neurons, while *α*-syn positive neurites are also seen in the neuropil.

#### 3.2.3. Olfactory Bulb Neurodegeneration

The olfactory bulb pathology deserves special attention because large segments of the world population are exposed to a myriad of toxic substances on a daily basis that have the potential for harming the olfactory system and penetrating the brain via the olfactory epithelium (OE) [68–71]. Extreme instances of such exposures in the USA include the massive dust cloud following the September 11, 2001, terrorist attack in New York City, smoke and debris from wildfires, exposures to airborne herbicides and pesticides in farming communities, and pollutants from vehicle exhaust and manufacturing enterprises in major metropolitan areas. The issue is very important because olfactory dysfunction is among the earliest “preclinical” features of AD and PD, occurring in *∼*90% of early onset cases [72–76]. 

**Figure 6:**
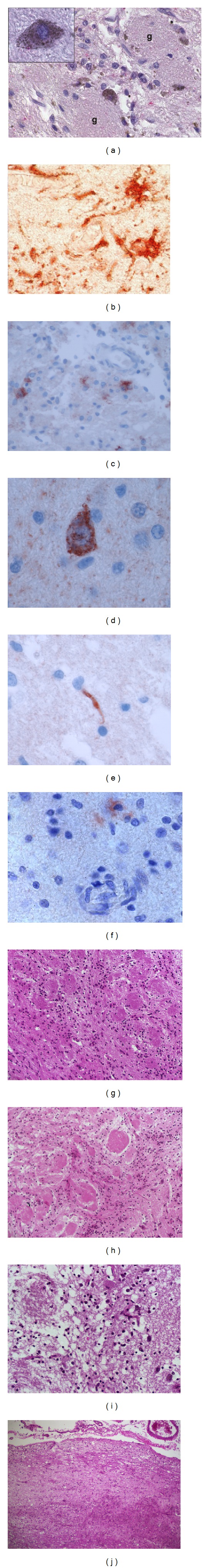
(a) Fourteen year old MCMA boy with abundant particulate material in neurons in the glomerular region. The insert shows a close-up of one such neuron with abundant particles and positive red cytoplasmic stain for A*β*42. A*β*42 immunohistochemistry and hematoxilin counterstain. (b) Reactive astrocytes are seen in the olfactory bulbs of MC children and teens. This is a 14 y old MCMA boy with reactive olfactory bulb astrocytes strongly staining for GFAP. GFAP immunohistochemistry with red product. (c) Olfactory bulb in an 11 y old MCMA boy APOE 3/3. Numerous neurons display positive cytoplasmic granular staining. *α*-Synuclein with red product. (d) A close-up of an olfactory bulb neuron with abundant *α*-Synuclein. (e) A close-up of a Lewy neurite is seen. *α*-Synuclein with red product. (f) Eleven year old MCMA male with *β*-amyloid 1–42 in olfactory bulb neurons. A*β*42 immunohistochemistry and hematoxilin counterstain. (g) This is the olfactory bulb of a control 20 year old male from a low polluted city. The glomerular structures are organized and exhibit normal cellular components. H&E. (h) In contrast, this is the olfactory bulb of an 11 year old MC boy APOE 3/3 with abnormal, loose and low cellular glomeruli. H&E. (i) Even more striking changes are seen in this 32 y old MC APOE 4/4 female. There are no remaining normal glomeruli, the few structures remaining are ill-defined with very few cells o no cells at all. It is expected this individual had significant olfactory deficits. H&E. (j) Same case as (i). The olfactory bulb shows extensive premature accumulation of corporae amylacea: glycoproteinaceous inclusions in astrocytic processes associated with astrocytic injury and gliosis. Premature accumulation of corpora amylacea plays an important role in the sequestration of toxic cellular metabolites. H&E.

 In MCMA residents, the severe pathological changes in the nasal respiratory epithelium go hand and hand with a marked decrease in olfactory neurons, significant changes in Bowman's glands, and pathologic Alzheimer and Parkinson's early stage changes within the olfactory bulbs (OBs) [77]. In one study comparing the OBs of 35 young MCMA residents versus 9 controls (20.8 ± 8.5 years) from a minimally polluted city, the MC residents exhibited significant amounts of particles in OB glomerular neurons (Figure 6(a)), while reactive astrocytes were prominent in young children (Figure 6(b)). Immunoreactivity to alpha-synuclein, a hallmark of Parkinson's disease was present in OB neurons of MCMA teens and young adult (Figures 6(c), 6(d), and 6(e)) [77]. While neuronal accumulation of A*β*42 was present in young children regardless of APOE genotype (Figure 6(f)). The basic laminar OB organization of the glomerular, external plexiform, mitral cell, internal plexiform, and granular cell layers of the controls were generally intact (Figure 6(g)). In contrast, ill-defined and fragmented organization of the olfactory bulb layers, including small acellular glomeruli characterized MCMA youngsters (Figure 6(h)). The changes were extreme in APOE 4 carriers (Figures 6(i) and 6(j)). The early olfactory deficits appear to be associated with the aforementioned presence of beta amyloid, alpha synuclein, particulate matter in glomerular structures and the massive distortion of the OB organization. 

### 3.3. The Role of the APOE Genotype in the Brain Effects of Air Pollution

The Apolipoprotein E (APOE) 4 polymorphism influences aging and age-related diseases including the risk for Alzheimer's disease [78–80]. The differential effects of ApoE isoforms on AD risk are given at least in part by the ability to affect A*β* aggregation and clearance in the brain, effects on synaptic plasticity, cell signaling, lipid transport and metabolism, and neuroinflammation [78]. APOE receptors influence both the CNS effects of APOE as well as A*β* metabolism and toxicity. The APOE 4 genotype (in contrast to APOE 3) is associated with oxidative stress and chronic inflammation [78]. In traumatic brain injury, APOE 4 carriers may be more predisposed to brain cellular damage as measured by S-100B and NSE concentrations [79]. APOE4 also influences plasma lipid concentrations, increases the risk of type 2 diabetes mellitus (particularly among obese subjects and smokers), conditions associated with high oxidative stress, neuroinflammation, and brain vascular damage [80]. In keeping with the current literature suggesting APOE 4 carriers have disadvantages in terms of brain repair, management of A*β* metabolism and toxicity and increased oxidative stress and chronic inflammation, we have shown MCMA APOE4 carriers have greater hyperphosphorylated tau and diffuse A*β* plaques versus E3 carriers (*Q* = 7.82, *P* = 0.005) [30]. This observation is important because based on our data, air pollution moderates the association between APOE genotype and neurodegenerative changes, that is, an APOE 4 carrier residing in a highly polluted environment will have an acceleration of neurodegenerative changes towards AD [35]. This information is critical when planning the neuroprotection of susceptible populations exposed to air pollutant components.

## 4. Compensatory Responses versus Neurotoxic and Neurodegenerative Changes. Friend or Foe?

In our pediatric studies, the early clinical olfactory deficits appear to be associated with the presence of misfolded proteins, reactive gliosis and vascular damage in the olfactory bulb and the frontal cortex [77]. There is no doubt the extensive olfactory bulb pathology likely affects OB proteins with critical functions [81]. Likewise, the prefrontal cortex differential regulation of key gene networks; that is, IL1, NF*κ*B, TNF, IFN, and TLRs are likely players in the significant cognitive deficits observed in children with no risk factors for neurological or cognitive deficits, other than their residency in a highly polluted megacity [29, 32, 33, 77]. In the same stream of thought, the central delay in the brainstem auditory evoked potentials and the significant white matter volumetric changes described after 1-year followup of MCMA versus control children could be related to the accumulation of abnormal proteins in key neuronal groups and the significant neuroinflammation involving both gray and white matter [30, 35, 67].

In view of the cognitive, olfactory, auditory, vestibular, and volumetric white matter changes described in exposed children, a series of critical questions arise:

1. What is the role of PM in the neuroinflammatory process described in highly exposed children?
2. What is the relationship between clinical and electrophysiological changes and the described neuropathology?
3. How to interpret the neuropathology hallmarks of AD and PD in a 10 year old child with no family history of neurological diseases?

Let us begin with the issue of particulate matter: Mexico City residents have been chronically exposed to concentrations of particulate matter above the USA standards for the last 26 years [1, 12, 13, 16]. A considerable fraction of the PM_2.5_ consists of organic compounds including biologic components from bacteria and fungi, and transition metals with neurotoxic properties [17–19]. Environmental endotoxins—from open field waste areas, waste water treatment plants, open sewer channels, and daily outdoor deposits of 500 metric tons of animal and human fecal material—are an important part of the organic portion of PM_2.5_.

Why is PM important for MCMA children? Because fine and ultrafine particles reach their brain by uptake through olfactory neurons and cranial nerves, trafficking of macrophage-like cells loaded with PM from the lung capillary bed to the systemic circulation, and by a direct transfer of ultrafine particles from the systemic circulation and/or red blood cells to brain endothelial cells [30, 35]. Our data and those of others suggest that exposure to PM can activate pathogen sensors, and that signaling by ROS can drive inflammatory processes [82–86]. Asbestos and silica activate the NALP 3 inflammasome and NALP3 deficient mice have a significant reduction of their lung inflammatory responses [41]. The innate immune system rapidly detects invading pathogenic microbes and eliminates them. We have shown an upregulation of 27/84 frontal inflammasome associated genes, including NOD-like receptors and proinflammatory caspases [30], so it is biologically plausible that PM with lipopolysaccharides (PM-LPS) initiates an inflammatory brain response. Toll-like receptors sense “extracellular microbes” (e.g., PM-LPS) and trigger anti-pathogen signaling cascades [84]. Both LPS responses and systemic inflammation are important for the understanding of how the sensing of “*microbial invaders*” could translate into signaling pathways that culminate in the transcriptional regulation of immune responsive genes and how the activation of inflammasomes [84] could be a contributing factor for CNS inflammatory responses. The inflammasome activation results in caspase 1 activation leading to processing and secretion of proinflammatory cytokines like IL1*β* to engage innate immune defenses [86]. Indeed, this pathway is clearly active in MCMA children: the activation of inflammasomes turns on the protease caspase-1. Caspase-1 cleaves prointerleukin-1*β* into an active form. We have repeatedly shown IL-1*β* in frontal cortex, olfactory bulb, hippocampus, and the dorsal vagal complex is upregulated in highly exposed children, dogs and mice compared to low pollution controls [30, 35, 87]. There is a clear need for better understanding of the role of inflammasome activation in urban children's brains and the defense against *pathogens *that do not really exist (only components of them, e.g., PM-LPS), and neuroinflammation. This is of particular importance as neuroprotective strategies are being explored. 

The relationship between clinical and electrophysiological changes and the described neuropathology is of deep interest to pediatricians working in polluted urban centers. We mentioned olfaction deficits and abnormal UPSIT (University of Pennsylvania Smell Identification Test) scores present in 35.5% of the MCMA teens versus 12% of age matched controls [77]. Moreover, highly exposed APOE 4 carriers failed 2.4 ± 0.54 of the 10 UPSIT items identified in one study as being most strongly related to AD [88], while APOE 2/3 and 3/3 subjects failed only 1.36 ± 0.16 such items (*P* = 0.01). The olfactory bulb neuropathology associated with urban exposures is very similar to the one described in early stages of AD and PD [89–96]. 

The central delayed brainstem auditory evoked potentials (BAEPs), auditory impairment and vestibular dysfunction could relate to the extensive brainstem inflammation with accumulation of *β* amyloid and alpha synuclein in key olfactory nuclei [67]. Neurodegenerative changes in the dorsal motor nucleus of the vagus, the nucleus of the solitary tract, arcuate nucleus, raphe midline, and extra-raphe medial and lateral tegmental neurons [67] are similar to the PD stages I and II of Braak et al. [90, 91, 96]. 

It is difficult to establish the association between cognitive deficits, frontal tau hyperphosphorylation, and amyloid-*β* diffuse plaques in the absence of cognitive and brain MRI data in the demised children. However, we have shown a strong relationship between residency, brain structural changes and cognitive deficits [32]. MCMA children with WMH (+) are responding to the air pollution exposures with white and grey matter volume increases in temporal, parietal, and frontal regions and better cognitive performance compared to WMH negative children [32]. WMH in elderly people are associated with clinical symptoms related to disruption of fiber tracts, cognitive impairment risk, cerebral ischemia, neurodegeneration, cardiovascular, and metabolic diseases [97–106]. WMH partially identify underlying white matter pathology and may be associated with widespread white matter changes, the novel concept of white matter hyperintensities penumbra [107]. Disruption of fiber tracts in the developing brain could result in cortical cholinergic and monoaminergic deafferentation and impact attention, emotion and goal-directed behavior [99]. The characterization of WMH in young urbanites is critical and knowledge about the complex modulation of cytokines and chemokines in the setting of air pollution are important because they may shed light into the etiopathogenesis of well-characterized risk factors for neurodegeneration, vascular, and cognitive disorders and disability [106, 107].

A difficult question to answer is how to interpret the early neuropathology hallmarks of Alzheimer's and Parkinson's diseases in children with no family history of neurological diseases. 

In the Alzheimer's brain, tau is abnormally hyperphosphorylated and it is aggregated into paired helical filaments forming neurofibrillary tangles, a histopathological hallmark of the disease [108]. Tau phosphorylation could be protective (e.g., hibernation) or toxic (e.g., hyperphosphorylation and aggregation of tau) [109]. Hyperphosphorylated tau in epitopes characteristic of AD has been identified by immunohistochemistry in 62.5% of APOE 4 and 33% of APOE 3 young MCMA carriers [30]. 

Is tau phosphorylation in children detrimental or protective in the setting of severe air pollution? [109–114]. The aggregation of HP tau species has been proposed to represent a compensatory neuronal response against oxidative stress and to serve at least initially as a protector against cell death [111, 113]. The tau protective or toxic function could be related to different conformational molecular changes [109]. The formation of tangles is a quick process as it was demonstrated by De Calignon et al. [112] using *in vivo* multiphoton imaging in living tau transgenic mice. Caspase activation precedes tangle formation by hours to days, tangles form quickly but persist apparently indefinitely, thus cleavage of tau is enough to cause misfolding of tau followed by nucleation and recruitment of additional tau molecules to the neuronal cell body. Is our description of HP*τ* in MCMA children's brains an isolated observation in the literature? The answer is no, Braak and Del Tredeci [93] examined 42 young brains (4–29 years) with a wide range of pathologies described pretangle HP*τ* using AT8 in 38/42 cases with no extracellular amyloid *β* protein deposition or neuritic plaques with the 4G8 antibody. Although these subjects were not healthy, there was no APOE genotyping or a recorded history of environmental exposures, we fully agree with Braak and Del Tredeci [93] that these findings may indicate Alzheimer's disease-related pathological process leading to neurofibrillary tangle formation start quite early, before puberty or in early young adulthood.

There are very few arguments about the role of abnormal tau hyperphosphorylation in AD, related tauopathies and under experimental conditions [108, 109, 114–118]. A subject to be explored in air pollution animal models ought to be the characterization of the HP*τ* and if indeed represents a compensatory neuronal response against oxidative stress. At this time, however, we are of the opinion that given the factors (chronic oxidative stress, neuroinflammation, presence of nanosize particles in critical brain units and anatomical regions) potentially accounting for the aggregation of tau, tau phosphorylation could represent an early sensor of oxidative stress with all the subsequent detrimental effects if the exposure persist.

Likewise, A*β*42 is capable of aggregation and misfolding leading to progressive neurodegeneration that develops insidiously over a lifetime. A key issue has to be addressed in this scenario: APOE4 carriers not only have HP*τ*, but also exhibit significant numbers of A*β* 6E10 diffuse plaques (*P* = 0.005) in comparison to APOE 3 carriers. Recent work by Cerf et al. [119] suggests that APOE4 strongly stabilizes A*β* oligomers, the pathological species responsible for AD; thus we suggest APOE4 carriers are potentially at a higher risk of developing AD if residing in a highly polluted environment. This information is critical given that *∼*18% of the MCMA population carries an APOE 4 allele [30]. 

Alpha-synuclein aggregation is associated to the pathogenesis of Parkinson's disease and exposure to a myriad of environmental agents, including agrochemicals increases the PD risk [120, 121]. Mitochondrial dysfunction and oxidative stress constitute key PD pathogenic events. Alpha-synuclein prevents cytochromec release and apoptosis through inhibition of the MAPK signaling pathway, suggesting that endogenous concentrations of *α*-synuclein confer resistance to oxidative stress downstream of free radical production and scavenging [122]. Recent evidence also suggests misfolded *α*-synuclein directly activates microglia inducing the production and release of the proinflammatory cytokine, TNF-*α*, and increasing antioxidant enzyme expression [123]. Béraud et al. emphasized the importance of protein misfolding, oxidative stress, and inflammation in PD as a potential locus for the development of novel therapeutics focused on induction of the Nrf2-directed antioxidant pathway and inhibition of protein misfolding [123].

It is important to note that *α*-synuclein in MCMA children is present in key regions associated with PD pathology including olfactory bulb, the midbrain, and the lower sections of the brainstem, for example, the medulla oblongata [67, 77]. MCMA teens exhibit already olfactory disturbances [77] and autonomic dysfunction (syncope in MCMA children personal communication of Dr. Maricela Franco-Lira), the latter severe enough to require pediatric care. The issue of MCMA children already showing symptoms seen in the premotor stages of PD has to be well thoughtout [73, 74] given the neurodegenerative process begins earlier in the olfactory bulb and lower brain stem and the fact there is a delay of several decades between the onset of dopaminergic denervation and the appearance of motor signs [96]. There is no question olfactory dysfunction is an early “preclinical” sign of Parkinson's disease [73, 74]. Damage to cholinergic, serotonergic, and noradrenergic components of the olfactory pathway likely involved to explain the olfactory dysfunction [73, 74]. The presence of up-regulated inflammatory cytokines, *α*-synuclein- and HP*π*-related olfactory bulb pathology in young highly exposed children is an ominous sign possibly associated with a number of other nonmotor symptoms related to PD, such as dysautonomia and sleep disturbances. Epidemiological studies addressing nonmotor PD symptoms in highly exposed young urbanites are warranted.

### 4.1. Looking Forward and Limitations

Despite controversy regarding the mechanistic pathways involved in the CNS damage associated with exposure to air pollutants, specifically fine and ultrafine particles of diverse origin, animal models and tissue culture studies have greatly improved our understanding of the mechanistic processes [39, 41, 42, 48–52, 58–66, 69–71]. We are looking forward to bridging the gap between early neuroinflammation and neurodegeneration observed in childhood and early adulthood and experimental air pollution animal models. There is a strong need for collaborations between those who investigate humans and those who study experimental animal models to derive therapies that may be neuroprotective. There is also a need for looking into the neuropathology in diverse populations residing in megacities across the globe and sharing the results of the investigations. This is critical since the responses to air pollutants depend not only on the components of air pollution and concentrations, but also on the genetic background of the exposed populations and on a large list of environmental factors including dietary risk factors, obesity, alcohol intake, and lifelong experiences for example, educational and occupational attainment [124]. Our results are potentially limited by the characteristics of the air pollutants in MCMA and the populations we are studying, namely ethnic groups with a complex admixture of ancestral populations as seen with Mexican mestizos. Nevertheless, the significant differences in clinical and neuropathology findings between high and low pollution exposed subjects warrants extensive investigations in exposed populations from countries around the world. 

## 5. Summary

MCMA children experience a chronic, intense state of oxidative stress resulting from lifelong exposures to a severely polluted environment. Children exhibit an early brain imbalance in genes involved in oxidative stress, inflammation, innate and adaptive immune responses, cell proliferation and apoptosis. Neuroinflammation, endothelial activation, endothelial cell hyperplasia, the attachment of white blood cells to the endothelial damaged walls with the reduction of the lumen vessel, high blood concentrations of endothelin-1, and the breakdown of the BBB clearly contribute to cognitive impairment and pathogenesis and pathophysiology of neurodegenerative states [125, 126]. Environmental and genetic factors play a key role in their CNS responses as evidenced by the acceleration of neurodegenerative AD pathology in children carrying an APOE 4 allele.

The neuronal accumulation of misfolded proteins in exposed children coincides with the anatomical distribution observed in the early stages of both AD and PD with early clinical evidence of olfactory and cognitive deficits, brain volumetric changes, white matter hyperintense lesions, altered brainstem evoked auditory potentials and autonomic disbalance. There is a complex modulation of cytokines and chemokines influencing structural and volumetric brain responses and cognitive deficits.

We contend that misfolding of critical proteins could be a defensive early response to the sustained systemic and CNS inflammation. However, the sustained oxidative stress associated with dysregulated inflammation, both systemic and in the CNS contribute to the establishment of neurodegenerative processes with clinical early counterparts. We strongly support the contention that the nasal (olfactory and trigeminal), cardiorespiratory and gastrointestinal (vagus) pathways-along with the systemic direct transport of particles to the brain and the dysregulated systemic inflammation are critical in explaining the brain pathology in highly exposed MCMA children. Moreover, these children are at risk of developing Alzheimer's and Parkinson's diseases as adults. 

We have a 50-year window of opportunity between the early brain changes observed in children and the time when the patient with mild cognitive impairment or dementia will show up at the neurologist's door. Facing the current pediatric clinical and pathology evidence is imperative if we are aiming our efforts to identify and mitigate environmental factors that influence AD and PD pathogenesis.

One thing is clear: early implementation of neuroprotective measures to ameliorate or stop the inflammatory and neurodegenerative processes in children is warranted [43, 87]. Identification of biomarkers associating systemic inflammation to brain growth is also critical for detecting children at higher risk for cognitive deficits and neurodegeneration. 

It is important to remember there is a severe and woeful deficit of progress in the development of both AD and PD-modifying therapy [127, 128]. Since fine and ultrafine PM likely play a key role in the development of neuroinflammation and neurodegeneration, it is very noteworthy that in the US alone, as of December 2012, more than 74 million people are being exposed to concentrations of PM_2.5_ above the 2006 standards (PM_2.5_ annual standard of 15 *μ*g/m^3^) [129]. An appeal to research supporting institutions may be made to strongly invest in defining the CNS pathology associated with exposure to air pollutants in children and young adults and as Castellani and Perry suggested, consider a systems biology approach and an early preventive pathway [128]. 

Epidemiological, cognitive, and mechanistic studies into the association between air pollution exposures and the development of CNS damage in children are of pressing importance for public health and quality of life. 

## References

[1] Molina LT, Molina MJ (2004). Improving air quality in megacities: Mexico City case study. *Annals of the New York Academy of Sciences*.

[2] Chen B, Kan H (2008). Air pollution and population health: a global challenge. *Environmental Health and Preventive Medicine*.

[3] Parrish DD, Zhu T (2009). Clean air for megacities. *Science*.

[4] Bloom DE (2011). 7 Billion and counting. *Science*.

[5] Rodrigues R, Smith MA, Wang X (2012). Molecular neuropathogenesis of Alzheimer’s disease: an interaction model stressing the central role of oxidative stress. *Future Neurology*.

[6] Nunomura A, Moreira PI, Castellani RJ (2012). Oxidative damage to RNA in aging and neurodegenerative disorders. *Neurotoxicity Research*.

[7] Hirsch EC, Jenner P, Przedborski S Pathogenesis of Parkinson’s disease.

[8] Block ML, Calderón-Garcidueñas L (2009). Air pollution: mechanisms of neuroinflammation and CNS disease. *Trends in Neurosciences*.

[9] Oberdörster G, Elder A, Rinderknecht A (2009). Nanoparticles and the brain: cause for concern?. *Journal of Nanoscience and Nanotechnology*.

[10] Brook RD, Rajagopalan S, Pope CA (2010). American Heart Association Council on Epidemiology and Prevention, Council on the Kidney in Cardiovascular Disease, and Council on Nutrition, Physical Activity and Metabolism. Particulate matter air pollution and cardiovascular disease: an update to the scientific statement from the American Heart Association. *Circulation*.

[11] Kampfrath T, Maiseyeu A, Ying Z (2011). Chronic fine particulate matter exposure induces systemic vascular dysfunction via NADPH oxidase and TLR4 pathways. *Circulation Research*.

[12] Molina LT, Kolb CE, de Foy B (2007). Air quality in North America’s most populous city—overview of the MCMA-2003 campaign. *Atmospheric Chemistry and Physics*.

[13] Molina LT, Madronich S, Gaffney JS (2010). An overview of the MILAGRO 2006 Campaign: Mexico City emissions and their transport and transformation. *Atmospheric Chemistry and Physics*.

[14] Evans J, Levy J, Hammitt J, Molina LT, Molina MJ (2002). Health benefits of air pollution control. *Air Quality in the Mexico Megacity: An Integrated Assessment*.

[15] Ezcurra E, Mazari-Hiriart M (1996). Are megacities viable? A cautionary tale from Mexico City. *Environment*.

[16] Bravo-Alvarez H, Torres-Jardَn R, Fenn M, de Bauer L, Hernández T (2002). Air pollution levels and trends in the México City metropolitan area. *Urban Air Pollution and Forest: Resources at Risk in the Mexico City Air Basin*.

[17] Osornio-Vargas AR, Bonner JC, Alfaro-Moreno E (2003). Proinflammatory and cytotoxic effects of Mexico City air pollution particulate matter in vtro are dependent on particle size and composition. *Environmental Health Perspectives*.

[18] Rosas Pérez I, Serrano J, Alfaro-Moreno E (2007). Relations between PM_10_ composition and cell toxicity: a multivariate and graphical approach. *Chemosphere*.

[19] Querol X, Pey J, Minguillَn MC (2008). PM speciation and sources in Mexico during the MILAGRO-2006 Campaign. *Atmospheric Chemistry and Physics*.

[20] SMA-GDF (Secretaria del Medio Ambiente del Gobierno del Distrito Federal) Sistema de Monitoreo Atmosférico. Secretaría del Medio Ambiente. http://www.calidadaire.df.gob.mx/calidadaire/index.php.

[21] Tang T, Hurraß, Gminski R, Mersch-Sundermann V (2012). Fine and ultrafine particles emitted from laser printers as indoor air contaminants in German offices. *Environmental Science and Pollution Research*.

[22] Fröhlich E, Roblegg E (2012). Models for oral uptake of nanoparticles in consumer products. *Toxicology*.

[23] Atkinson RW, Carey IM, Kent AJ, van Staa TP, Anderson HR, Cook DG (2013). Long-term exposure to outdoor air pollution and incidence of cardiovascular disease. *Epidemiology*.

[24] Zhang ZF, Yu SZ, Zhou GD (1988). Indoor air pollution of coal fumes as a risk factor of stroke, Shanghai. *American Journal of Public Health*.

[25] Villeneuve PJ, Johnson JY, Pasichnvk D, Lowes J, Kirkland S, Rowe BH (2012). Short-term effects of ambient air pollution on stroke: who is most vulnerable?. *Science of the Total Environment*.

[26] Calderón-Garcidueňas L, Vincent R, Mora-Tiscareňo A (2007). Elevated plasma endothelin-1 and pulmonary arterial pressure in children exposed to air pollution. *Environmental Health Perspectives*.

[27] Calderón-Garcidueñas L, Mora-Tiscareño A, Fordham LA (2003). Respiratory damage in children exposed to urban pollution. *Pediatric Pulmonology*.

[28] Calderón-Garcidueñas L, Macias-Parra M, Hoffmann HJ (2009). Immunotoxicity and environment: immunodysregulation and systemic inflammation in children. *Toxicologic Pathology*.

[29] Calderón-Garcidueñas L, Mora-Tiscareňo A, Ontiveros E (2008). Air pollution, cognitive deficits and brain abnormalities: a pilot study with children and dogs. *Brain and Cognition*.

[30] Calderón-Garcidueñas L, Kavanaugh M, Block ML (2012). Neuroinflammation, hyperphosphorilated tau, diffuse amyloid plaques and down- regulation of the cellular prion protein in air pollution exposed children and adults. *Journal of Alzheimer Disease*.

[31] Calderón-Garcidueñas L, Serrano-Sierra A, Torres-Jardón R The impact of environmental metals in young urbanites’ brains.

[32] Calderón-Garcidueñas L, Mora-Tiscareón A, Styner M (2012). White matter hyperintensities, systemic inflammation, brain growth and cognitive functions in children exposed to air pollution. *Journal of Alzheimer's Disease*.

[33] Calderón-Garcidueñas L, Torres-Jardón R (2012). Air pollution, socioeconomic status and children’s cognition in megacities: the Mexico City Scenario. *Frontiers in Psychology*.

[34] Rivest S, Lacroix S, Vallières L, Nadeau S, Zhang J, Laflamme N (2000). How the blood talks to the brain parenchyma and the paraventricular nucleus of the hypothalamus during systemic inflammatory and infectious stimuli. *Proceedings of the Society for Experimental Biology and Medicine*.

[35] Calderón-Garcidueñas L, Solt AC, Henríquez-Roldán C (2008). Long-term air pollution exposure is associated with neuroinflammation, an altered innate immune response, disruption of the blood-brain barrier, ultrafine particulate deposition, and accumulation of amyloid *β*-42 and *α*-synuclein in children and young adults. *Toxicologic Pathology*.

[36] Saunders NR, Liddelow SA, Dziegielewska KM (2012). Barrier mechanisms in the developing brain. *Frontiers in Psychology*.

[37] Heumann D, Roger T (2002). Initial responses to endotoxins and Gram-negative bacteria. *Clinica Chimica Acta*.

[38] Bonner JC, Rice AB, Lindroos PM (1998). Induction of the lung myofibroblast PDGF receptor system by urban ambient particles from Mexico City. *American Journal of Respiratory Cell and Molecular Biology*.

[39] Yang EJ, Kim S, Kim JS, Choi JH (2012). Inflammasome formation and IL1*β* release by human blood monocytes in response to silver nanoparticles. *Biomaterials*.

[40] Guarda G, Dostert C, Staehli F (2009). T cells dampen innate immune responses through inhibition of NLRP1 and NLRP3 inflammasomes. *Nature*.

[41] Dostert C, Pétrilli V, van Bruggen R, Steele C, Mossman BT, Tschopp J (2008). Innate immune activation through Nalp3 inflammasome sensing of asbestos and silica. *Science*.

[42] Morris M, Li L (2012). Molecular mechanisms and pathological consequences of endotoxin tolerance and priming. *Archivum Immunologiae Et Therapiae Experimentalis*.

[43] Villarreal-Calderón R, Dale G, Delgado-Chavez R (2012). Intra-city differences in cardiac expression of inflammatory genes and inflammasomes in young urbanites: a pilot study. *Journal of Toxicologic Pathology*.

[44] Calderón-Garcidueñas L, Engle R, Mora-Tiscareño A (2011). Exposure to severe urban pollution influences cognitive outcomes, brain volume and systemic inflammation in clinically healthy children. *Brain Cognition*.

[45] Calderón-Garcidueñas L, Azzarelli B, Acuña H (2002). Air pollution and brain damage. *Toxicologic Pathology*.

[46] Calderón-Garcidueñas L, Maronpot RR, Torres-Jardon R, Henríquez-Roldán C, Schoonhoven R, Acuña-Ayala H (2003). DNA damage in nasal and brain tissues of canines exposed to air pollutants is associated with evidence of chronic brain inflammation and neurodegeneration. *Toxicologic Pathology*.

[47] Chambers JK, Mutsuga M, Uchida K, Nakayama H (2011). Characterization of A*β*pN3 deposition in the brains of dogs of various ages and other animal species. *Amyloid*.

[48] Yu CH, Song GS, Yhee JY (2011). Histopathological and immunohistochemical comparison of the brain of human patients with Alzheimer’s disease and the brain of aged dogs with cognitive dysfunction. *Journal of Comparative Pathology*.

[49] Cotman CW, Head E (2008). The canine (dog) model of human aging and disease: dietary, environmental and immunotherapy approaches. *Journal of Alzheimer’s Disease*.

[50] Campbell A, Araujo JA, Li H, Sioutas C, Kleinman M (2009). Particulate matter induced enhancement of inflammatory markers in the brains of apolipoprotein E knockout mice. *Journal of Nanoscience and Nanotechnology*.

[51] Levesque S, Surace MJ, McDonald J, Block ML (2011). Air pollution and the brain: subchronic diesel exhaust exposure causes neuroinflammation and elevates early markers of neurodegenerative disease. *Journal of Neuroinflammation*.

[52] Levesque S, Taetzsch T, Lull ME (2011). Diesel exhaust activates and primes microglia: air pollution, neuroinflammation and regulation of dopaminergic neurotoxicity. *Environmental Health Perspectives*.

[53] Rivest S (2009). Regulation of innate immune responses in the brain. *Nature Reviews Immunology*.

[54] Simard AR, Rivest S (2004). Role of inflammation in the neurobiology of stem cells. *NeuroReport*.

[55] Fassbender K, Walter S, Kühl S (2004). The LPS receptor (CD14) links innate immunity with Alzheimer’s disease. *The FASEB Journal*.

[56] Nguyen MD, Julien JP, Rivest S (2002). Innate immunity: the missing link in neuroprotection and neurodegeneration?. *Nature Reviews Neuroscience*.

[57] Kim WK, Alvarez X, Fisher J (2006). CD163 identifies perivascular macrophages in normal and viral encephalitic brains and potential precursors to perivascular macrophages in blood. *American Journal of Pathology*.

[58] Wu J, Wang C, Sun J, Xue Y (2011). Neurotoxicity of silica nanoparticles: brain localization and dopaminergic neurons damage pathways. *ACS Nano*.

[59] Sharma HS, Sharma A (2012). Neurotoxicity of engineered nanoparticles from metals. *CNS & Neurological Disorders*.

[60] Trickler WJ, Lantz SM, Schrand AM (2012). Effects of copper nanoparticles on rat cerebral microvessel endothelial cells. *Nanomedicine*.

[61] Win-Shwe TT, Fujimaki H (2011). Nanoparticles and neurotoxicity. *International Journal of Molecular Sciences*.

[62] Ho YS, Yang X, Yeung SC (2012). Cigarette smoking accelerated brain aging and induced pre-Alzheimer-like neuropathology in rats. *PLoS One*.

[63] Lucchini RG, Dorman DC, Elder A, Veronesi B (2012). Neurological impacts from inhalation of pollutants and the nose-brain connection. *Neurotoxicology*.

[64] Qin L, Wu X, Block ML (2007). Systemic LPS causes chronic neuroinflammation and progressive neurodegeneration. *GLIA*.

[65] Hartz AMS, Bauer B, Block ML, Hong JS, Miller DS (2008). Diesel exhaust particles induce oxidative stress, proinflammatory signaling, and P-glycoprotein up-regulation at the blood-brain barrier. *The FASEB Journal*.

[66] MohanKumar SM, Campbell A, Block M, Veronesi B (2008). Particulate matter, oxidative stress and neurotoxicity. *Neurotoxicology*.

[67] Calderón-Garcidueñas L, D’Angiulli A, Kulesza RJ (2011). Air pollution is associated with brainstem auditory nuclei pathology and delayed brainstem auditory evoked potentials. *International Journal of Developmental Neuroscience*.

[68] Tjälve H, Henriksson J, Tallkvist J, Larsson BS, Lindquist NG (1996). Uptake of manganese and cadmium from the nasal mucosa into the central nervous system via olfactory pathways in rats. *Pharmacology and Toxicology*.

[69] Tjalve H, Henriksson J (1999). Uptake of metals in the brain via olfactory pathways. *NeuroToxicology*.

[70] Dorman DC, Struve MF, Marshall MW, Parkinson CU, James RA, Wong BA (2006). Tissue manganese concentrations in young male rhesus monkeys following subchronic manganese sulfate inhalation. *Toxicological Sciences*.

[71] Wang Y, Wang B, Zhu MT (2011). Microglial activation, recruitment and phagocytosis as linked phenomena in ferric oxide nanoparticle exposure. *Toxicology Letters*.

[72] Doty RL (2009). Symposium overview: do environmental agents enter the brain via the olfactory mucosa to induce neurodegenerative diseases. *Annals of the New York Academy of Sciences*.

[73] Doty RL (2012). Olfactory dysfunction in Parkinson’s disease. *Nature Reviews Neurology*.

[74] Doty RL (2012). Olfaction in Parkinson’s disease and related disorders. *Neurobiology of Disease*.

[75] Schofield PW, Ebrahimi H, Jones AL, Bateman GA, Murray SR (2012). An olfactory “stress test” may detect preclinical Alzheimer’s disease. *BMC Neurology*.

[76] Rahayel S, Frasnelli J, Joubert S (2012). The effects of Alzheimer’s disease and Parkinson’s disease on olfaction: a meta-analysis. *Brain Research*.

[77] Calderón-Garcidueñas L, Franco-Lira M, Henríquez-Roldán C (2010). Urban air pollution: influences on olfactory function and pathology in exposed children and young adults. *Experimental and Toxicologic Pathology*.

[78] Holtzman DM, Herz J, Bu G (2012). Apolipoprotein e and apolipoprotein e receptors: normal biology and roles in Alzheimer disease. *Cold Spring Harbor Perspectives in Medicine*.

[79] Olivecrona Z, Koskinen LO (2012). The release of S-100B and NSE in severe traumatic head injury is associated with ApoE 4. *Acta Neurochirurgica*.

[80] Chaudhary R, Likidlilid A, Peerapatdit T (2012). Apolipoprotein E gene polymorphism: effects on plasma lipids and risk of type 2 diabetes and coronary artery disease. *Cardiovascular Diabetology*.

[81] Fernández-Irigoyen J, Corrales FJ, Santamaria E (2012). Proteomic atlas of the human olfactory bulb. *Journal of Proteomics*.

[82] Schroder K, Sagulenko V, Zamoshnikova A (2012). Acute lipopolysaccharide priming boosts inflammasome activation independently of inflammasome sensor induction. *Immunobiology*.

[83] Lamkanfi M, Dixit VM (2012). Inflammasomes and their roles in health and disease. *Annual Review of Cell and Developmental Biology*.

[84] Martinon F (2010). Signaling by ROS drives inflammasome activation. *European Journal of Immunology*.

[85] Hanamsagar R, Hanke ML, Kielian T (2012). Toll-like receptor (TLR) and inflammasome actions in the central nervous system. *Trends in Immunology*.

[86] Latz E (2010). The inflammasomes: mechanisms of activation and function. *Current Opinion in Immunology*.

[87] Villarreal-Calderon R, Torres-Jardón R, Palacios-Moreno J (2010). Urban air pollution targets the dorsal vagal complex and dark chocolate offers neuroprotection. *International Journal of Toxicology*.

[88] Tabert MH, Liu X, Doty RL (2005). A 10-item smell identification scale related to risk for Alzheimer’s disease. *Annals of Neurology*.

[89] Braak H, Braak E (1991). Neuropathological stageing of Alzheimer-related changes. *Acta Neuropathologica*.

[90] Braak H, Del Tredici K, Rüb U, de Vos RAI, Jansen Steur ENH, Braak E (2003). Staging of brain pathology related to sporadic Parkinson’s disease. *Neurobiology of Aging*.

[91] Braak H, Rüb U, Gai WP, Del Tredici K (2003). Idiopathic Parkinson’s disease: possible routes by which vulnerable neuronal types may be subject to neuroinvasion by an unknown pathogen. *Journal of Neural Transmission*.

[92] Braak H, Thal DR, Ghebremedhin E, Del Tredici K (2011). Stages of the pathologic process in Alzheimer disease: age categories from 1 to 100 years. *Journal of Neuropathology & Experimental Neurology*.

[93] Braak H, Del Tredici K (2011). The pathological process underlying Alzheimer’s disease in individuals under thirty. *Acta Neuropathologica*.

[94] Jellinger K, Braak H, Braak E, Fischer P (1991). Alzheimer lesions in the entorhinal region and isocortex in Parkinson’s and Alzheimer’s diseases. *Annals of the New York Academy of Sciences*.

[95] Jellinger KA (2012). Interaction between pathogenic proteins in neurodegenerative disorders. *Journal of Cellular and Molecular Medicine*.

[96] Meissner WG (2012). When does Parkinson's disease begin? From prodromal disease to motor signs. *Revue Neurologique*.

[97] Jefferson AL, Massaro JM, Wolf PA (2007). Inflammatory markers are associated with total brain volume: the Framingham Heart Study. *Neurology*.

[98] Jefferson AL, Massaro JM, Beiser AS (2011). Inflammatory markers and neuropsychological functioning: the Framingham Heart Study. *Neuroepidemiology*.

[99] Bohnen NI, Miiller MLTM, Kuwabara H, Constantine GM, Studenski SA (2009). Age-associated leukoaraiosis and cortical cholinergic deafferentation. *Neurology*.

[100] Bohnen NI, Albin RL (2011). White matter lesions in Parkinson disease. *Nature Reviews Neurology*.

[101] Silbert LC, Howieson DB, Dodge H, Kaye JA (2009). Cognitive impairment risk: white matter hyperintensity progression matters. *Neurology*.

[102] Brickman AM, Zahra A, Muraskin J (2009). Reduction in cerebral blood flow in areas appearing as white matter hyperintensities on magnetic resonance imaging. *Psychiatry Research*.

[103] Murray ME, Senjem ML, Petersen RC (2010). Functional impact of white matter hyperintensities in cognitively normal elderly subjects. *Archives of Neurology*.

[104] Gouw AA, Seewann A, van der Flier WM (2011). Heterogeneity of small vessel disease: a systematic review of MRI and histopathology correlations. *Journal of Neurology, Neurosurgery and Psychiatry*.

[105] Bunce D, Anstey KJ, Cherbuin N (2010). Cognitive deficits are associated with frontal and temporal lobe white matter lesions in middle-aged adults living in the community. *PLoS ONE*.

[106] Wallin A, Fladby T (2010). Do white matter hyperintensities on MRI matter clinically?. *British Medical Journal*.

[107] Maillard P, Fletcher E, Harvey D (2011). White matter hyperintensity penumbra. *Stroke*.

[108] Wang JZ, Xia YY, Grundke-Iqbal I, Iqbal K Abnormal hyperphosphorylation of tau: sites, regulation and molecular mechanism of neurofibrillary degeneration.

[109] Avila J, León-Espinosa G, García E, Garcia-Escudero V, Hernández F, Defelipe J (2012). Tau phosphorilation by GSK3 in different conditions. *International Journal of Alzheimer's Disease*.

[110] Bonda DJ, Castellani RJ, Zhu X (2011). A novel perspective on Tau in Alzheimer’s disease. *Current Alzheimer Research*.

[111] Lee HG, Perry G, Moreira PI (2005). Tau phosphorylation in Alzheimer’s disease: pathogen or protector?. *Trends in Molecular Medicine*.

[112] De Calignon A, Fox LM, Pitstick R (2010). Caspase activation precedes and leads to tangles. *Nature*.

[113] Buée L, Troquier L, Burnouf S (2010). From tau phosphorylation to tau aggregation: what about neuronal death?. *Biochemical Society Transactions*.

[114] Avila J (2010). Intracellular and extracellular tau. *Frontiers in Neuroscience*.

[115] Hanger DP, Wray S (2010). Tau cleavage and tau aggregation in neurodegenerative disease. *Biochemical Society Transactions*.

[116] Cui B, Zhu L, She X (2012). Chronic noise exposure causes persistence of tau hyperphosphorilation and formation of NFT tau in the rat hippocampus and prefrontal cortex. *Experimental Neurology*.

[117] Lénárt N, Szegedi V, Juhász G (2012). Increased Tau phosphorilation and impaired presynaptic function in hypertriglyceridemic ApoB-100 transgenic mice. *PLOS ONE*.

[118] Lee CW, Shih YS, Wu SY, Yang T, Lin C, Kuo YM Hypoglycemia induces tau hyperphosphorilation.

[119] Cerf E, Gustot A, Goormaghtigh E, Ruysschaert JM, Raussens V (2011). High ability of apolipoprotein E4 to stabilize amyloid-*β* peptide oligomers, the pathological entities responsible for Alzheimer’s disease. *The FASEB Journal*.

[120] Silva BA, Breydo L, Fink AL, Uversky VN Agrochemicals, *α*-synuclein and Parkinson’s disease.

[121] Perfeito R, Cunha-Oliveira T, Rego AC (2012). Revisiting oxidative stress and mitochondrial dysfunction in the pathogenesis of Parkinson’s disease-resemblance to the effect of amphetamine drugs of abuse. *Free Radical Biology & Medicine*.

[122] Musgrove RE, King AE, Dickson TC a-Synuclein protects neurons from apoptosis downstream of free-radical production through modulation of the MAPK signaling pathway.

[123] Béraud D, Hathaway HA, Trecki J Microglial activation and antioxidant responses induced by the Parkinson’s disease protein a-synuclein.

[124] Stern Y (2012). Cognitive reserve in aging and Alzheimer’s disease. *Lancet Neurology*.

[125] Jian H, Yi-Fang W, Qi L, Xiai-Song H, Gui-Yun Z Cerebral blood flow and metabolic changes in hippocampal regions of a modified rat model with chronic cerebral hypoperfusion.

[126] Roher AE, Debbins JP, Malek-Ahmadi M (2012). Cerebral blood flow in Alzheimer’s disease. *Journal of Vascular Health and Risk Management*.

[127] De la Torre JC (2012). A turning point for Alzheimer’s disease?. *Biofactors*.

[128] Castellani RJ, Perry G (2012). Pathogenesis and disease-modifying therapy in Alzheimer’s disease: the flat line of progress. *Archives of Medical Research*.

[129] http://www.epa.gov/oaqps001/greenbk/.

